# Deep learning for predicting refractive error from multiple photorefraction images

**DOI:** 10.1186/s12938-022-01025-3

**Published:** 2022-08-08

**Authors:** Daoliang Xu, Shangshang Ding, Tianli Zheng, Xingshuai Zhu, Zhiheng Gu, Bin Ye, Weiwei Fu

**Affiliations:** 1 School of Biomedical Engineering (Suzhou), Division of Life Sciences and Medicine, University of Science and Technology of China, Suzhou, China; 2 Suzhou Institute of Biomedical Engineering and Technology, Chinese Academy of Science, Suzhou, China

**Keywords:** Deep learning, Refractive error, Convolutional neural network, Photorefraction, Image processing, Myopia

## Abstract

**Background:**

Refractive error detection is a significant factor in preventing the development of myopia. To improve the efficiency and accuracy of refractive error detection, a refractive error detection network (REDNet) is proposed that combines the advantages of a convolutional neural network (CNN) and a recurrent neural network (RNN). It not only extracts the features of each image, but also fully utilizes the sequential relationship between images. In this article, we develop a system to predict the spherical power, cylindrical power, and spherical equivalent in multiple eccentric photorefraction images.

Approach

First, images of the pupil area are extracted from multiple eccentric photorefraction images; then, the features of each pupil image are extracted using the REDNet convolution layers. Finally, the features are fused by the recurrent layers in REDNet to predict the spherical power, cylindrical power, and spherical equivalent.

**Results:**

The results show that the mean absolute error (MAE) values of the spherical power, cylindrical power, and spherical equivalent can reach 0.1740 D (diopters), 0.0702 D, and 0.1835 D, respectively.

**Significance:**

This method demonstrates a much higher accuracy than those of current state-of-the-art deep-learning methods. Moreover, it is effective and practical.

## Background

Refractive error is a type of vision problem that makes it difficult to see clearly; the incidence of refractive error is increasing, especially in the case of myopia [1]. The number of people with myopia worldwide is approximately 2.6 billion, 312 million of which are under the age of 19, and this number is continuously rising [2]. Early refractive error detection plays an essential role in controlling the development of myopia, and currently there are two main refractive error detection methods: autorefraction and eccentric photorefraction. Eccentric photorefraction has the characteristics of simplicity and speed, and it requires less cooperation from individuals, making it suitable for large-scale or infant-vision screening. However, it is lower in accuracy [3, 4].

In recent years, deep learning has achieved much in several fields. Deep learning can automatically extract features without manual rules setting [5]. In medicine, deep learning has been applied in the diagnosis of brain diseases [6], retinal diseases [7], COVID-19 [8], and breast cancer [9]. Many researchers have used deep-learning methods for refractive detection. Some researchers have used fundus images to detect refractive errors. Varadarajan et al. [10] first proposed a deep-learning method for refractive error detection in 2018. Tan et al. [11] developed a deep-learning system to predict refractive error and myopic macular degeneration from color fundus images. Manivannan et al. [12] used wide-field fundus images to estimate refractive errors and axial lengths. Some researchers, such as Chun et al. [13] and Fu et al. [14] have used eccentric photorefraction images taken by mobile phones. Other special methods have also been used to detect refractive errors, for example, from posterior segment optical coherence tomography (OCT) or ocular appearance images [15, 16]. However, the results of the aforementioned studies showed a lack of precision. The best mean absolute error (MAE) value of the spherical equivalent (SE) was only 0.56 D, which was obtained by Manivannan et al. [12], and the cylindrical power was not predicted; these results indicated that the related methods remain far from practical application.

There are two factors that can account for the low accuracy of the aforementioned studies: algorithms and images. Currently, most of the algorithms use the popular neural networks, which are proposed for tasks, such as image classification, object detection, and semantic segmentation, instead of unusual tasks, such as diopter detection. In addition, these studies often employ the fundus images, eccentric photorefraction images taken by mobile phones, OCT images, and other ocular images. Fundus and OCT images are mainly used for fundus disease detection, and mobile phones only have a fixed eccentric distance and meridian direction. Neither method is suitable for high-precision diopter detection.

Therefore, a refractive error detection network (REDNet) is proposed for the detection of refractive error in multiple eccentric photorefraction images with different meridian directions. First, a convolutional neural network (CNN) with few parameters and high precision is employed to extract the features of six images, and then a recurrent neural network (RNN) is used to fuse the feature sequences through sequence processing. The spherical power, cylindrical power, and spherical equivalent are predicted with high accuracy using our method.

## Results

### Evaluation index

In the training process of the network, the MAE, defined in Eq. (1), was used as the primary evaluation index:

1 $$ {\text{MAE}} = \frac{1}{N}\sum\limits_{i = 1}^{N} {\left| {(y_{i} - \mathop {y_{i} }\limits^{ \wedge } )} \right|} . $$

This evaluation index is simple to calculate and can speed-up the training process. To compare the proposed method with existing methods of deep learning for refraction detection and evaluate the performance of our algorithm more specifically, we also used accuracy as an evaluation index. If the predicted value was within 0.5 D of the true value, we considered the prediction to be correct.

### Performance of the proposed feature extractor

Our proposed feature extractor was compared with a selection of traditional CNNs: VGG16 [17], ResNet18 [18], Xception [19], and mini-Xception [20]. Mini-Xception was inspired by Xception and is a lightweight network for facial expression recognition. A fully connected network with no activation function was added after the feature extraction layer of our network to output the predicted value of the diopter, thus forming a complete neural network. The neural network was trained using the eccentric photorefraction image in one direction as the input and the diopter value in the corresponding direction as the output. The single-orientation diopter was calculated using the spherical power (*S*), cylindrical power (*C*), and the cylindrical axis (*A*) as follows:

2 $$ D_{\alpha } = C\sin^{2} A + S $$

where *D*_*α*_ is the diopter in the meridian direction of *α* [21]. The results are presented in Table 1 and show that our proposed network has a positive effect on the diopter prediction from a single image while having a relatively small number of parameters.

**Table 1:** Detection results for different network structures

Network	MAE (D)	Accuracy (%)	No. parameters
Vgg16	0.7916	45.41	19.19 M
ResNet18	0.8617	42.34	11.18 M
Xception	0.3204	82.35	7.24 M
Mini-Xception	0.3440	79.26	0.79 M
Ours	**0.2792**	**86.46**	**1.19 M**

Bold values indicate the best results

### Performance of the proposed feature fusion

After feature extraction, 6 vectors of length 256 were extracted, and the effects of four fusion methods were compared: addition, concatenation, adaptive feature fusion (AFF), and long short-term memory (LSTM). To ensure fairness of the comparison, that is, to ensure that the parameters of the four comparison methods are approximately the same, two fully connected layers were added after the addition, concatenation, and AFF methods, corresponding to two layers of the LSTM. The results in Table 2 indicate that using LSTM to fuse features yields an exceptional result in the prediction of spherical power, cylindrical power, and spherical equivalent.

**Table 2:** Experimental results for different feature fusion methods

Methods	MAE (D)	Accuracy (%)	MAE (D)	Accuracy (%)	MAE (D)	Accuracy (%)
	Spherical component		Cylindrical component		Spherical equivalent	
Addition	0.2818	83.06	0.1210	96.39	0.2593	85.98
Concatenation	0.3663	74.02	0.1590	96.59	0.3196	78.19
AFF	0.2443	87.05	0.0761	96.40	0.2109	89.00
LSTM	**0.1740**	**89.50**	**0.0702**	**96.70**	**0.1835**	**89.38**

Bold values indicate the best results

### Activation function

According to the theory of eccentric photorefraction, the diopter is mainly obtained from the grayscale change in the pupil image in the meridian direction. However, actual pupil images often have occlusions due to eyelashes and the existence of Purkinje images in the center of the cornea [22], as shown in Fig. 6(g). Therefore, ReLU6 was selected as our activation function because the grayscale change in the area of interest should be filtered to exclude eyelashes and Purkinje images. The gradient values of the eyelashes and Purkinje images were relatively large, and this relatively large response should be suppressed for accurate results. The ReLU6 activation function is highly suitable for this task, and it is simple to calculate and fast to train. We compared the performance of several common activation functions, such as sigmoid, ELU [23], Swish [24], leaky ReLU [25], ReLU, and ReLU6 [19]. The results are presented in Table 3 and indicate that the ReLU6 obtained the best MAE and accuracy, and a higher training speed.

**Table 3:** Experimental results for different activation functions

Activation function	MAE (D)	Accuracy (%)	Time/step (ms)
Sigmoid	0.3636	75.85	20
ELU	0.3072	83.15	21
Swish	0.2874	84.78	22
Leaky ReLU	0.2843	85.00	20
ReLU	0.2792	86.46	20
ReLU6	**0.2703**	**87.03**	**20**

Bold values indicate the best results

#### Performance in predicting high myopia

**Fig. 1:**
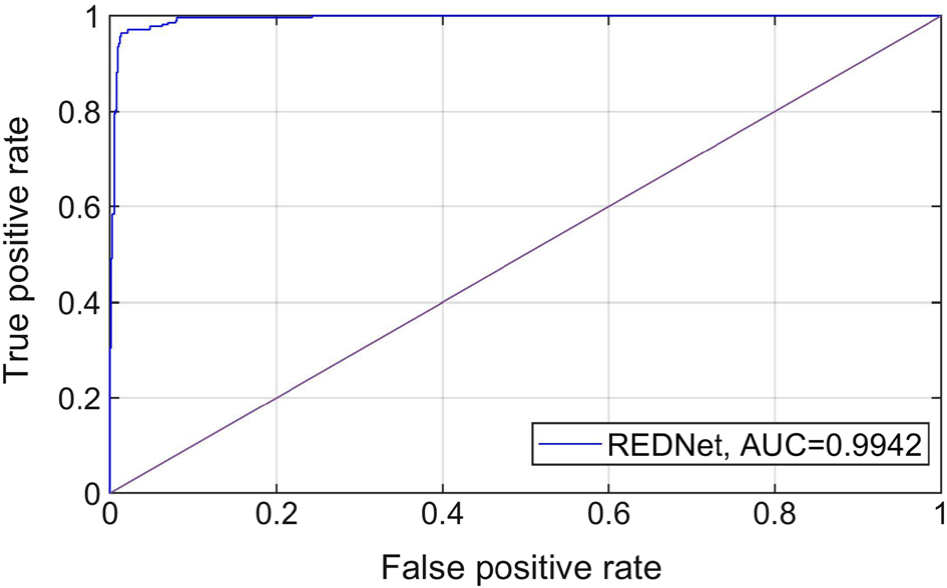
ROC curve and AUC value

Meanwhile, the performance of REDNet was evaluated in the prediction of high myopia using the receiver operating characteristic (ROC) curve, area under the curve (AUC) value, accuracy, specificity, and sensitivity. For evaluation, the diopter value was considered to represent the probability of high myopia. Fig. 1 shows the prediction performance of the proposed model. The results indicate that the proposed model has a significantly high AUC of 0.9942 [95% confidence interval(95% CI), 99.34–99.50%], accuracy of 97.13% (95% CI 96.93–97.34%), specificity of 97.19% (95% CI 96.95–97.43%), and sensitivity of 96.97% (95% CI 96.63–97.31%). This is not surprising because our model was designed to predict precise diopter value, and predicting high myopia is relatively simple.

### Visualization

**Fig. 2:**
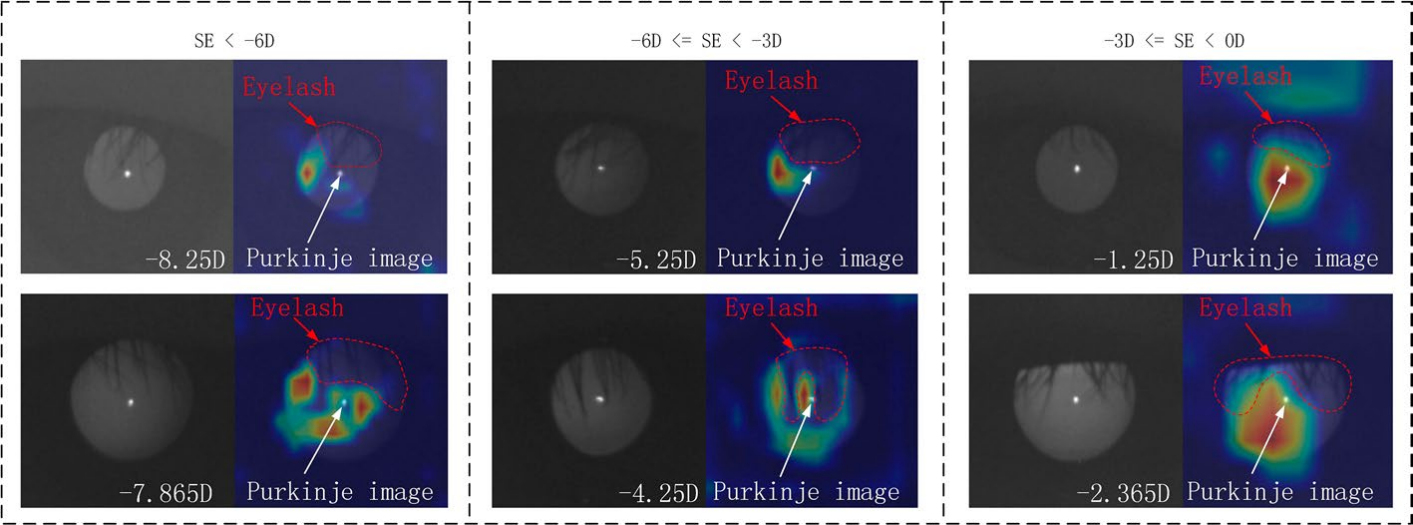
Visualization heat map of different refractive error

Gradient-weighted class activation mapping (Grad-CAM) [26] was used to explain the proposed neural network. The heat map drawn by Grad-CAM can detail the key areas emphasized by the neural network in the learning process, allowing us to determine whether the neural network has learned meaningful features. The heat map indicates that the key positions considered by the network are all in the pupil area (Fig. 2), which is consistent with the theory of eccentric photorefraction. In particular, our model avoids the region blocked by eyelashes and pays less attention to the regions of the Purkinje image, which are considered as noise for refractive detection; this demonstrates that our model obtains useful features.

### Validation of LSTM

This study also verified the importance of LSTM in improving network performance. We constructed and named two additional neural networks: REDNet-SimpleRNN that uses a simple RNN unit without gates, and REDNet-N that neither uses an RNN nor gates. The experimental results of the 3 networks on the spherical equivalent are listed in Table 4.

**Table 4:** Experimental results for the other two networks

Networks	RNN	Gate	MAE (D)	Accuracy (%)
REDNet-N			0.2890	84.32
REDNet-SimpleRNN	√		0.3428	80.04
REDNet	√	√	**0.1835**	**89.38**

Bold values indicate the best results

The results indicated that REDNet-N performed better than REDNet-SimpleRNN because of the long-term dependence problem of RNN, which means that the output result was only based on the last few sequences and previous sequences were ignored. However, the result of REDNet-N was the output of 6 sequences that were processed through the fully connected network, which involved the context relationship between sequences. Moreover, the results of REDNet were significantly superior, which indicated that the LSTM with gates solved the problem of long-term dependency, and the contextual information between the six sequences was effectively extracted; thus, the lowest MAE and highest accuracy was obtained.

## Discussion

Compared with other classical neural networks, the neural network based on the proposed feature extractor improved the prediction of single-orientation diopter from a single image with a smaller number of parameters. We demonstrated that the proposed feature extractor could effectively extract feature information, including unidirectional diopters, for unidirectional eccentric photorefraction images.

Our feature fusion method used an LSTM and performed better than addition, concatenation, and AFF. The results also demonstrated that the LSTM could effectively utilize the features containing six-direction diopter information and extract contextual relationships among the feature sequences; it could also effectively predict the spherical power, cylindrical power, and spherical equivalent.

The experimental results yielded MAE values of 0.1740, 0.0702, and 0.1835 D for spherical power, cylindrical power, and spherical equivalent, respectively. Additionally, the accuracy was much higher than current state-of-the-art deep-learning methods (0.56 D). The proposed method can predict the cylindrical power, and its accuracy is very close to automatic optometry. In addition, we can measure both eyes simultaneously; the object does not need to be fixed, and the operation is very simple and fast. Therefore, our method can be practically applied to large-scale vision screening and can play an important role in preventing and controlling myopia.

However, the number of subjects were relatively small, and the subjects were not distributed across all age groups and races, which affects the generalization of the model. This could be addressed with large-scale data collection in the future. Our images were obtained based on the theory of eccentric photography optometry. During the acquisition process, the distance between people and camera must be 1 m, which is relatively inconvenient in actual application. This limitation could be addressed by improving the method in the future.

## Conclusion

This paper proposed the REDNet, a neural network for refractive error detection that not only extracts the features of each image, but also fully utilizes the contextual relationship between images. The refractive error prediction method proposed in this study demonstrated high accuracy, which is superior to current deep learning-based methods, with the capability of predicting spherical power, cylindrical power, and spherical equivalent. In contrast, current deep-learning methods can only predict the spherical equivalent. However, the problems of generalization and distance limit the practical applicability of our method. Therefore, we will improve our method to tackle the aforementioned problems in future work.

## Methods

**Fig. 3:**
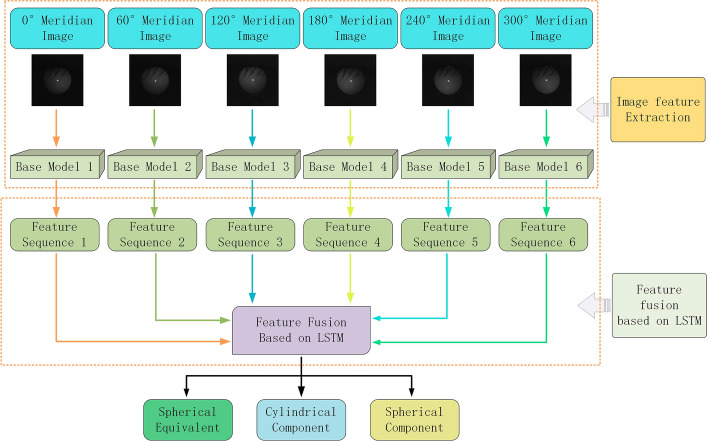
Overall structure of the proposed REDNet

The prediction of spherical power is relatively simple because all the required information about spherical power is included in a single image. However, cylindrical power cannot be predicted from a single image, as it is calculated by the difference in diopters in different meridian directions. In this study, we proposed REDNet, outlined in Fig. 3. First, 6 CNNs with the same structure were used to extract the features of six images, which were then fused into feature sequences. Next, we employed an RNN to process the sequence information to effectively fuse the features and predict the spherical power, cylindrical power, and spherical equivalent. The method proposed in this study included the following four steps: image acquisition and preprocessing, base model construction, feature fusion, and network training.

### Data acquisition and preprocessing

#### Data acquisition

All experiments were conducted indoors because the large amount of infrared light present outside would have affected the images we took. There were no specific requirements for the indoor conditions and the near-infrared (NIR) band-pass filter was able to ignore extra light.

**Fig. 4:**
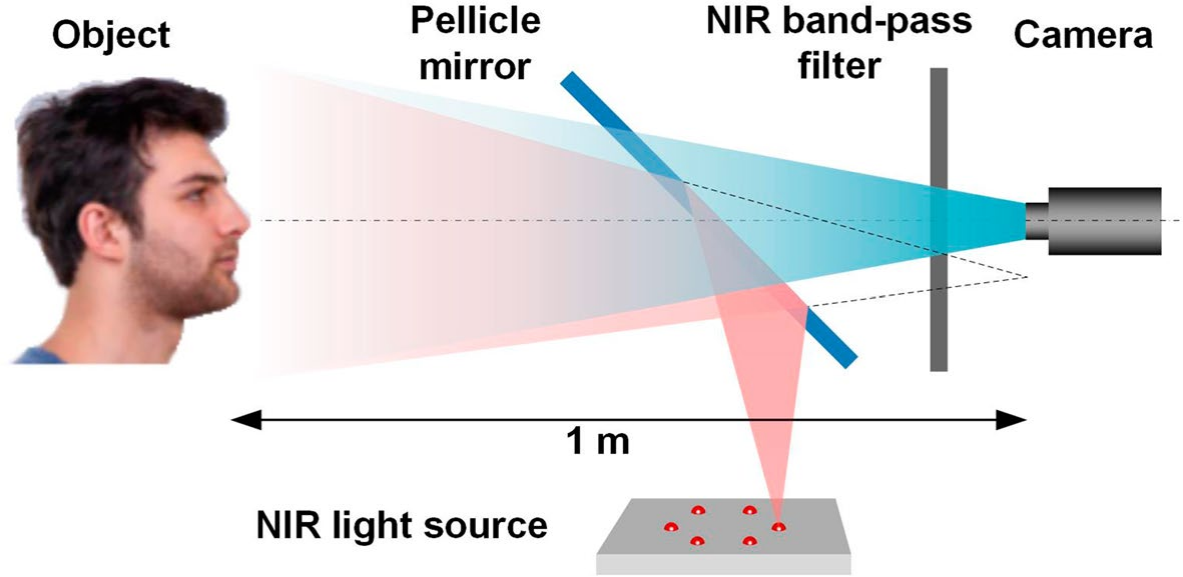
Method of data collection

The method of data collection is shown in Fig. 4. Infrared light emitted from the NIR light source (850 nm in wavelength), was used to illuminate the face through a Pellicle mirror [27]. The reflected light from the face passed through the NIR band-pass filter (800–900 nm) and was captured by the camera (Basler ace 2). Six face images in meridian directions (0°, 60°, 120°, 180°, 240°, and 300°) were obtained using NIR light sources at different locations [28]. The distance between the camera and the human eye was 1 m, and the distance between the light source and the human eye was also 1 m. The subject was not required to remain in a fixed position and was allowed to move slightly. The measurement process took approximately 15–30 s to complete.

A professional optometrist operated a TOPCON RM-800 automatic optometer to obtain the diopter using conventional optometry. During the measurements, the subject's chin was placed on the bracket to adjust the distance between the lens and the human eye. One eye was measured three times and the results were averaged. Because only one eye could be measured at a time, we switched to the other eye to continue the measurements. The subject was required to remain fixed throughout the process. The total measurement time was approximately 90–120 s.

#### Data preprocessing

**Fig. 5:**
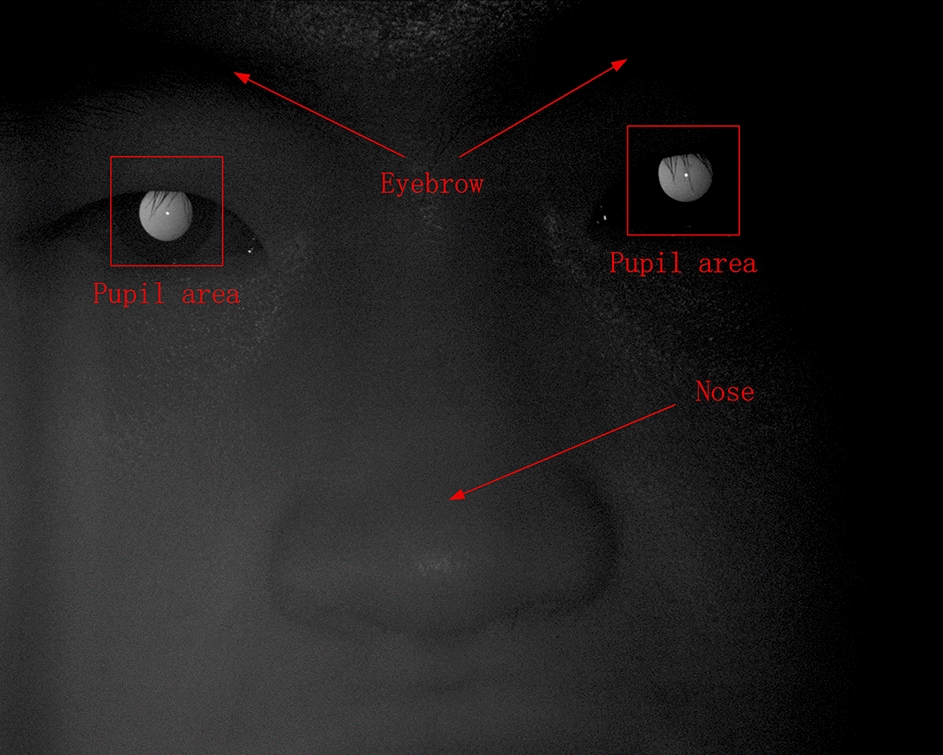
Example of a captured face image

An example of a captured face image is shown in Fig. 5. Because only the pupil region is related to the refraction, the rest of the face outside the pupil, such as the eyebrows and nose, should be removed. This is the purpose of image preprocessing. In this image, the brightness on the left side of the pupil is slightly higher than on the right, and this is the basis for our diopter prediction.

Six images of human pupils were used for prediction, and they only differed in terms of diopter information. However, in the process of image collection, the movement of the subject caused the pupils to be in different positions in the image; hence, data preprocessing was required. Data preprocessing was performed as follows: First, six images 1920 × 1080 in size of human faces including their pupils, were obtained. Then, the position of the pupil was located through threshold segmentation and template matching. Next, circle fitting for the pupil was performed, with the center of the circle as the center of the image. Finally, the pupil image was cropped to a size of 128 × 128; in the resulting 6 images, the pupil was approximately in the same position.

**Fig. 6:**
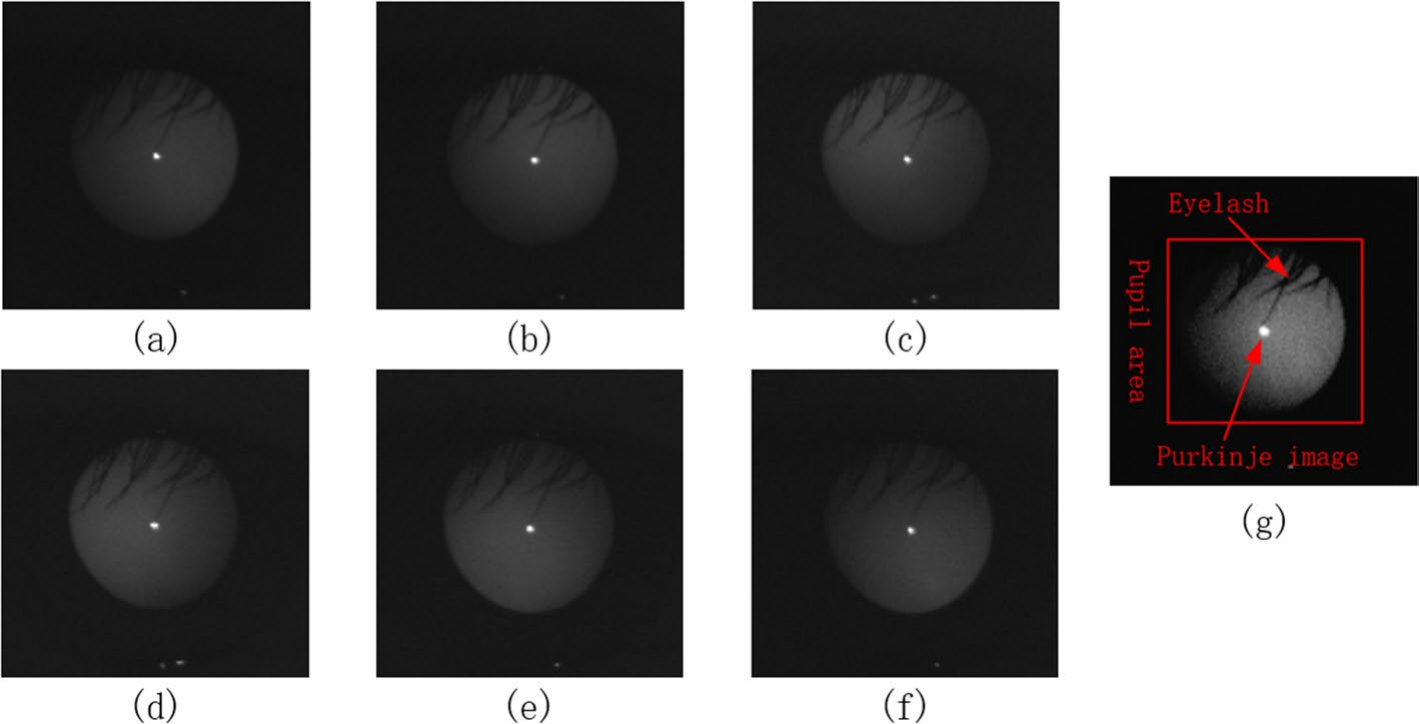
Eccentric photorefraction images of the same pupil with a meridian direction of **a** 0°, **b** 60°, **c** 120°, **d** 180°, **e** 240°, **f** 300°, and **g** 0° after contrast stretching

Each group of facial images can be used to extract two groups of pupil images, with one group containing six meridian directions, as shown in Fig. 6. The diopter was obtained by evaluating the reflection type and position of the eccentric crescent in the pupil image [27, 28]. Figure 5(a) shows an eccentric photorefraction image with a meridian direction of 0°, where the pixel changes are unnoticeable. By contrast, Fig. 5(g) shows that in the pupil area of the human eye, the pixel gray value changes from high to low along the direction of 0° after contrast stretching. Although such changes are unnoticeable and there is no clear boundary for us to evaluate the type and position of the eccentric crescent, we used deep learning to extract such obscure features.

A total of 3103 sets of eccentric photorefraction images of faces were collected. The age distribution of subjects ranged from 18–56 years. The mean age was 31.54 years, and the median age was 37 years. The proportion of the age groups is 38.54, 34.77, and 26.69% for age groups 18–25, 25–40, and 40–56 years, respectively. All samples with eye diseases, such as cataract, glaucoma, macular deformation, or a history of surgery were excluded to ensure that the collected data were normal. After extracting the pupil area from the images through image preprocessing, a total of 6146 groups of pupil images were obtained. Images that were blurred due to an incorrect focus position were eliminated, leaving 6074 groups of images. Among them, 3907 groups were used for the training set, 1167 groups were used for the validation set, and 1000 groups were used for the test set. The distribution of the datasets is presented in Table 5.

**Table 5:** Data distribution before and after data cleaning

Characteristic	Before cleaning	After cleaning
Number of image groups	6146	6074
Severe myopia (SE < -6 D)	1708	1699
Moderate myopia (−6 D ≤ SE < -3 D)	2395	2378
Mild myopia (−3 D ≤ SE < 0 D)	1919	1879
Emmetropia and hyperopia (SE ≥ 0 D)	124	118

### Network architecture

In this section, we present the design concepts of CNNs and RNNs. A CNN extracts features from multiple directional images to obtain a feature sequence. An RNN is used to process the feature sequence and then fuses the features. Finally, the fused features are sent to the fully connected neural network to obtain the spherical power, cylindrical power, and spherical equivalent.

We separated the six pupil images for feature extraction because they came from six different facial images. Even after image preprocessing, the content within the same position in the different images was not exactly the same. If they were to be combined into a six-channel image, the information from the six channels is mixed after convolution, and the cylindrical power information could not be effectively predicted.

In the present study, the last layer of feature fusion of the CNN was used. The last layer was the result of the global average pooling of multi-channel feature maps, which not only contained sufficient information but also had a small number of parameters. Feature fusion was highly efficient in this case.

#### Feature sequence extraction

**Fig. 7:**
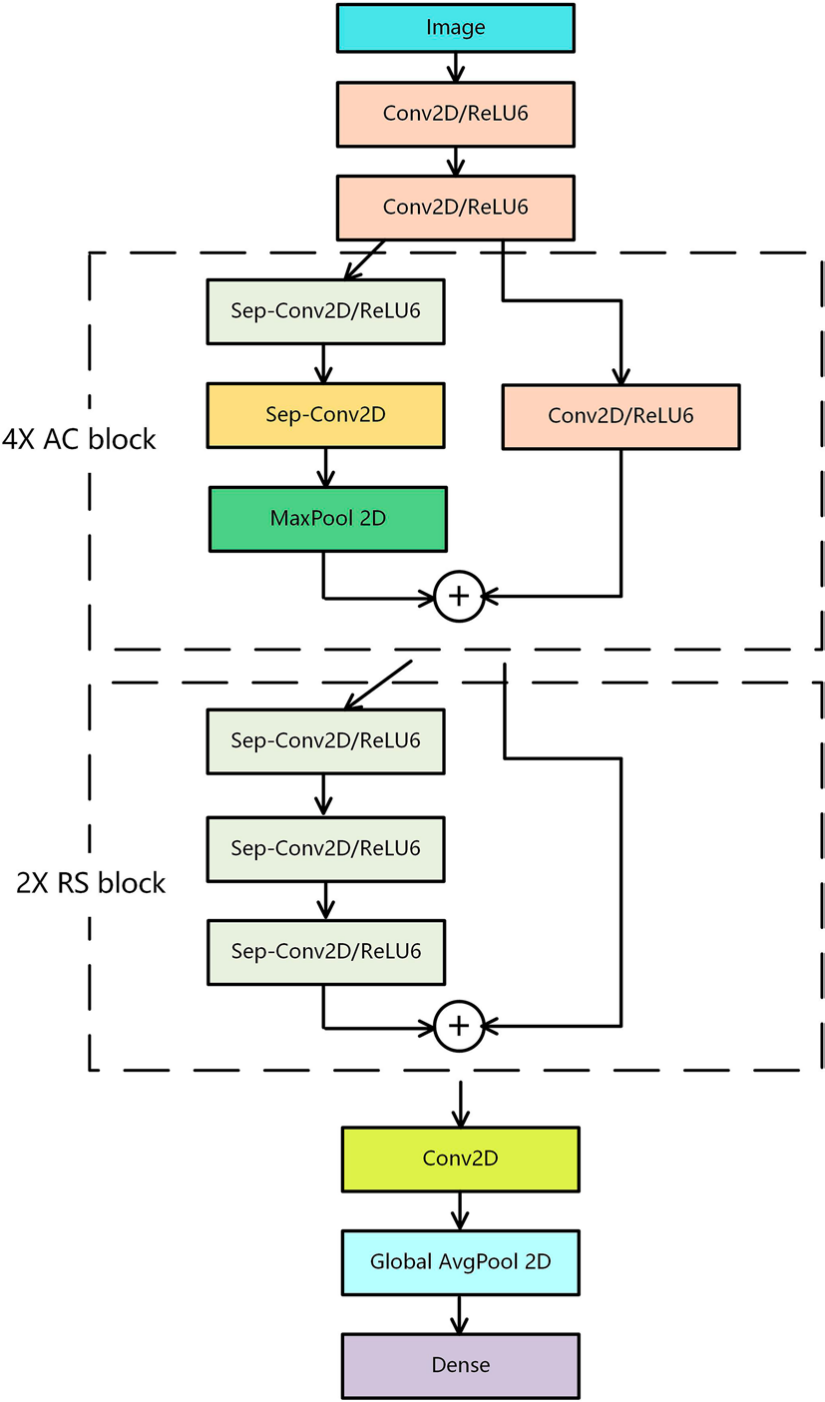
Structure of the constructed CNN

The CNN was designed to ensure that sufficient features can be extracted while having fewer parameters. The proposed network structure was inspired by the Xception network structure. The purpose of Xception was to reduce the number of parameters, but its original structure was still redundant. However, its use of residual modules and a depthwise separable convolution is useful for reducing the number of network parameters [19]. The network proposed in this study was composed of an automatic selection feature (ASF) block and depthwise separable residual (DSR) block. The network structure is shown in Fig. 7.

The residual connection directly connects the two-layer networks with gaps and prevents some convolutional layers from learning redundant features through identity mapping, thereby avoiding network redundancy and solving the problem of gradient disappearance in overly deep networks [18]. In the ASF block, a direct residual connection was not used; instead, a convolution was added to the position of the residual connection so that the network can automatically choose whether to go through two layers of depthwise separable convolution and maximum pooling or direct convolution features. In the DSR block, the residual connection was directly used, and the depthwise separable convolution of the three layers was directly connected, which avoids the problem of gradient disappearance and gradient explosion caused by too many convolution layers.

A depthwise separable convolution comprises depthwise and pointwise convolutions. The depthwise convolution uses a single convolutional filter for each feature map, and only calculates the spatial correlation within each feature map. The number of feature maps before and after the calculation was the same. Pointwise convolution projects the output of the depthwise convolution onto a new channel space and calculates the channel correlation of the feature maps. The number of output feature maps depends on the number of pointwise convolution kernels. Using depthwise separable convolutions decouples spatial and channel correlations while reducing the parameters of the network [29].

Global average pooling was used in the last layer. In some CNNs, such as VGG16 [17], the last few layers use fully connected neural networks, which make up a large part of the parameters. However, global average pooling can greatly reduce parameters and extract features that represent the whole, which satisfies the purpose of extracting features with CNNs.

#### Feature sequence fusion based on LSTM

Commonly used feature fusion methods in deep learning are feature map concatenation [30] or the direct addition of feature maps [18]; some researchers have also proposed AFF [31]. When the number of feature maps is large, concatenation increases the amount of computation and causes information redundancy. The direct addition of feature maps causes a discrimination conflict. AFF can automatically assign different weights to different feature maps and then add them together. Although AFF solves the problems of concatenation and addition, it is still unsuitable for this study.

In this study, we have six feature sequences that represent the diopter in six directions. The cylindrical power, which describes the diopter difference in various directions of the human eye, is obtained through the relationship between the diopters in the six directions. This means that the feature fusion focuses on the contextual relationship between feature sequences, whereas the purpose of the AFF is to work with one or several features, and the contextual relationship between sequences cannot be extracted. Therefore, we introduce an RNN to effectively learn the contextual relationship between the six feature sequences.

An LSTM is used to process feature sequences [32] and is a type of RNN that is usually applied to time sequences, such as in natural language processing. The LSTM has three gates: input, forget, and output gates. These three gates selectively store and output information and solve the problem of gradient disappearance and gradient explosion in a simple RNN [33]. These three gates also solve the long-term dependency problem of a simple RNN, which can only handle relatively close contextual information; this is important for the present study, as we had 6 sequences of equal importance. Hence, it would have been inappropriate to only focus on close contextual information.

After the CNN was constructed, 6 sequences were extracted from the six images, and the 6 sequences were sent to the RNN to predict the overall diopter.

### Network training

During the process of network training, the initial weights of the network are not random; if the initial weight of the network is given random parameters, the feature sequence extracted by the CNN is random, and the RNN has difficulty learning the relationship between the six sequences. Hence, the error cannot be effectively backpropagated into the CNN, and the cylinder cannot be accurately predicted. Therefore, an image from a single direction was taken as input and the diopter in a single direction was output. To pretrain the CNN, the pretrained parameters were taken as the initial weights, and then the overall training of the network was performed.

During training, we used the TensorFlow framework to implement our network model, the Adam optimization algorithm was used first for rapid gradient descent, and then the stochastic gradient descent optimization algorithm was used for fine-tuning [34]. The mean squared error was used as the loss function.

## Data Availability

The datasets used and/or analyzed during the current study are available from the corresponding author on reasonable request.
